# Quantitative Analysis of the Flavonoid Glycosides and Terpene Trilactones in the Extract of *Ginkgo biloba* and Evaluation of Their Inhibitory Activity towards Fibril Formation of β-Amyloid Peptide

**DOI:** 10.3390/molecules19044466

**Published:** 2014-04-10

**Authors:** Haiyan Xie, Jing-Rong Wang, Lee-Fong Yau, Yong Liu, Liang Liu, Quan-Bin Han, Zhongzhen Zhao, Zhi-Hong Jiang

**Affiliations:** 1 School of Chinese Medicine, Hong Kong Baptist University, Kowloon Tong, Kowloon, Hong Kong, China; 2 State Key Lab of Quality Research in Chinese Medicine, Macau Institute for Applied Research in Medicine and Health, Macau University of Science and Technology, Macau, China

**Keywords:** *Ginkgo biloba*, inhibitory activity, β-amyloid fibril formation, quantitative analysis, flavonoid glycoside, terpene trilactone

## Abstract

The standard extract of *Ginkgo* *biloba* leaves (EGb761) is used clinically in Europe for the symptomatic treatment of impaired cerebral function in primary degenerative dementia syndromes, and the results of numerous *in vivo* and *in vitro* studies have supported such clinical use. The abnormal production and aggregation of amyloid β peptide (Aβ) and the deposition of fibrils in the brain are regarded as key steps in the onset of Alzheimer’s Disease (AD), and the inhibition of Aβ aggregation and destabilization of the preformed fibrils represent viable approaches for the prevention and treatment of AD. Flavonoid glycosides and terpene trilactones (TTLs) are the two main components of EGb761 which represent 24 and 6% of the overall content, respectively. In our research, seven abundant flavonoid glycosides **1**–**7** were isolated from the extract of *Ginkgo* *biloba* leaves and characterized by spectroscopic analysis. Furthermore, an ultra-high performance liquid chromatography method was established for the simultaneous quantification of these seven flavonoids. The inhibitory activities of these flavonoids, as well as four TTLs, *i.e.*, ginkgolides A, B, and C and bilobalide (compounds **8**–**11**), were evaluated towards Aβ42 fibril formation using a thioflavin T fluorescence assay. It was found that three flavonoids **1**, **3** and **4** exhibited moderate inhibitory activities, whereas the other four flavonoids **2**, **5**, **6** and **7**, as well as the four terpene trilactones, showed poor activity. This is the first report of the inhibition of Aβ fibril formation of two characteristic acylated flavonoid glycosides **6**, **7** in *Ginkgo* leaves, on the basis of which the structure-activity relationship of these flavonoids **1**–**7** was discussed.

## 1. Introduction

Alzheimer’s disease (AD) is the main type of senile dementia, and is characterized by a range of pathological features, including the formation of senile plaques and neurofibrillary tangles. In recent years, there has been an increase in the number of reports identifying amyloid β peptide (Aβ) as a primary pathological factor in the occurrence of AD, with gradual changes in the balance between the state of Aβ production and clearance leading to the accumulation of aggregated Aβ. This accumulation of Aβ then triggers a series of complex reactions that ultimately lead to neuronal death and cognitive dysfunction [1].

The abnormal production and aggregation of Aβ and the deposition of fibrils in the brain are regarded as key steps in the onset of AD, and the development of therapeutic strategies aimed at preventing the aggregation of Aβ or promoting the destabilization of the preformed fibrils, therefore represent viable approaches for the prevention and treatment of AD [2].

EGb761 is the standard extract of the leaves of *Ginkgo* *biloba* which is currently being manufactured by several companies in Germany and France. On the grounds of proven clinical efficacy, EGb761 has been approved for the symptomatic treatment of impaired cerebral function in dementia syndromes by the German Federal Health Authority and for cerebral circulatory insufficiency and AD in France. The main indications for EGb761 are primary degenerative dementia, vascular dementia and mixed forms of dementia, which are characterized by several major symptoms, including memory loss, poor concentration, depression, vertigo, tinnitus, and headache. The approved daily dose of EGb761 is 120 to 240 mg in 2 or 3 doses, with doses of this type typically being recommended for at least 8 weeks. The results of a large number of *in vivo* and *in vitro* studies have provided strong evidence in support of the clinical use of EGb761 [3].

Flavonoid glycosides (FGs) and terpene trilactones (TTLs) are the two main groups of constituents in EGb761, which represent 24 and 6% of the total weight of EGb761, respectively. To identify the bioactive compounds in EGb761, we isolated the individual components of FGs from EGb and evaluated their inhibitory activities as well as single compounds of TTLs towards Aβ42 fibril formation using a thioflavin T (Th-T) fluorescence assay [4]. Using a variety of different chromatographic purification techniques, we isolated seven of the main FGs (compounds **1**–**7**) from EGb and elucidated their structures based on their HR-MS, ^1^H-NMR and ^13^C-NMR spectral data. We also developed an ultra-high performance liquid chromatography (UPLC)-UV method for the quantitative analysis of these compounds, which allowed for the quantification of these seven FGs as well as the four main TTL components (compounds **8**–**11**) in the extracts and preparations of *Ginkgo* *biloba* leaves. Finally, the activities of these flavonoids and terpene lactones were also investigated, on the basis of which the structure-activity relationship of these flavonoids **1**–**7** was discussed.

## 2. Results and Discussion

### 2.1. Isolation and Structural Elucidation of the FGs

**Figure 1:**
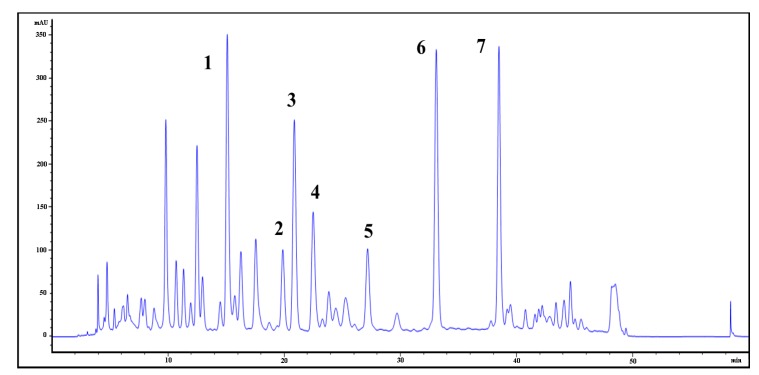
HPLC fingerprint of the total extract of the leaves of *Ginkgo biloba*.

Seven flavonoids **1**–**7** were isolated from the total extract of the leaves of *Ginkgo* *biloba*. The HPLC fingerprint of the total extract showed that these seven compounds accounted for 54% of the total peak area of the 34 peaks observed in the HPLC chromatogram (Figure 1).

**Figure 2:**
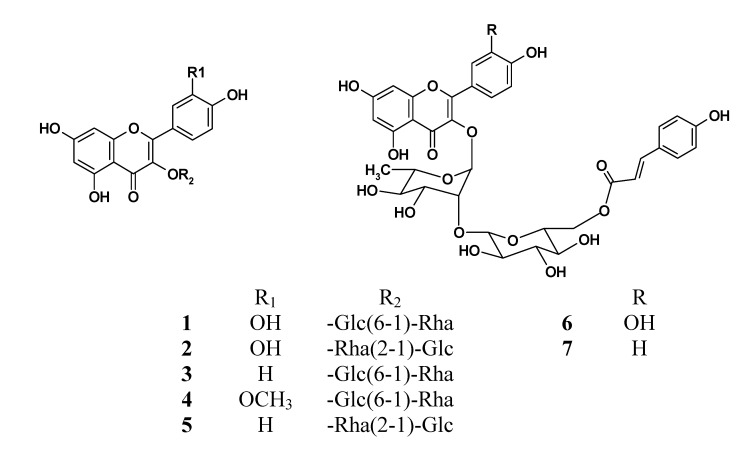
Structures of compounds **1**–**7**.

All seven compounds were obtained as yellow amorphous powders and their structures were determined based on a comparison of their MS and NMR spectral data with those in the literature [5,6,7,8]. Compounds **1**–**7** were identified as quercetin 3-*O*-β-D-rutinoside (**1**), quercetin 3-*O*-α-L-(β-D-glucopyranosyl)-(1,2)-rhamnopyranoside (**2**), kaempferol 3-*O*-β-D-rutinoside (**3**), isorhamnetin 3-*O*-β-D-rutinoside (**4**), kaempferol 3-*O*-α-L-(β-D-glucopyranosyl)-(1,2)-rhamnopyranoside (**5**), quercetin 3-*O*-α-(6′′′-p-coumaroyl glucopyranosyl-β-1,2-rhamnopyranoside) (**6**), and kaempferol 3-*O*-α-(6′′′-p-coumaroyl glucopyranosyl-β-1,2-rhamnopyranoside) (**7**), respectively (Figure 2).

### 2.2. Quantification of the FGs in Extracts and Preparations of Ginkgo biloba Leaves

#### 2.2.1. Method Validation

The regression equations, linearity, intra- and inter-day precision properties, and LOD and LOQ values of the seven analytes were determined using our UPLC-UV method. As shown in Table 1, satisfactory linearity levels were observed for compounds **1**–**7** (R^2^ ≥ 0.999). The LODs (S/N = 3) and LOQs (S/N = 10) of these analytes were less than 7.40 µg/mL and 24.75 µg/mL, respectively. The results listed in Table 2 showed that the intra- and inter-day variations were less than 1.63% (*n* = 5) and 3.65% (*n* = 5) for all of the analytes, respectively, demonstrating that this method exhibited a high level of precision for all of the analytes tested. The repeatability values (RSDs, *n* = 5) of compounds **1**–**7** were determined to be less than 1.94%, and the results of the recovery study showed that the average recovery rates of all seven compounds were in the range of 98.1%–102.8% while RSDs were all less than 2.08% (*n* = 7). Taken together, these results demonstrated that our UPLC-UV method was suitable for the subsequent quantitative analysis of the seven flavonoid glycosides.

**Table 1:** Linearity and sensitivity of compounds **1**–**7**.

Compounds	Regression Equation	Linear range (µg/mL)	*R^2^*	LOD (µg/mL)	LOQ (µg/mL)
1	Y = 9.1207x − 3.07	25.0–300.0	0.9999	2.33	8.09
2	Y = 6.4290x + 6.08	24.0–144.0	0.9995	4.44	14.66
3	Y = 11.370x − 4.72	12.5–150.0	0.9999	2.74	9.10
4	Y = 7.9820x − 17.96	25.0–150.0	0.9996	4.02	13.46
5	Y = 9.1149x − 5.52	25.0–150.0	0.9997	4.30	14.33
6	Y = 6.6509x + 4.18	25.0–150.0	0.9998	7.17	23.89
7	Y = 7.5831x − 3.76	25.0–150.0	0.9999	7.40	24.75

**Table 2:** Precision, repeatability and recovery of compounds **1**–**7**.

Compds.	Precision (*n* = 5)			Repeatability (*n* = 5)				Recovery (*n* = 7)		
	Intra-day RSD/%	Inter-day RSD/%		Concentration (µg/mg)	RSD/%				Mean	RSD/%	
**1**	0.53	1.29		6.92	1.67				102.8	1.65	
**2**	1.63	3.65		3.33	1.05				98.1	1.90	
**3**	0.67	1.78		3.74	1.02				99.8	0.68	
**4**	0.65	1.30		4.75	1.94				100.6	1.23	
**5**	0.42	1.09		2.61	1.11				99.9	1.52	
**6**	0.36	1.15		6.31	1.45				99.3	2.08	
**7**	0.54	0.39		4.78	1.15				100.3	1.61	

#### 2.2.2. Quantitative Analysis of Compounds **1**–**7** in the Extracts and Tablets of *Ginkgo biloba*

In this study, we collected a total of 11 samples for UPLC-UV analysis, including the extractsand botanical products of *Ginkgo* *bil*oba, *i.e.*, Tanakan^®^ and Ginaton^®^ tablets, which were made from EGb761. The details of these samples are listed in Table 3.

**Table 3:** Details of the samples subjected to quantitative analysis.

Sample	Description
S1	Extract, extracted from tablets tanakan® (made from EGb761 and manufactured by Beaufour Ipsen Industrie , Dreux, France and purchased from Watsons, HK)
S2	Extract, extracted from tablets tanakan® (made from EGb761 and manufactured by Beaufour Ipsen Industrie, Dreux, France and purchased from Tianjin, China)
S3	Extract, extracted from tablets Ginaton® (made from EGb761 and manufactured by Dr. Willmar Schwabe GmbH &Co. KG, Karlsruhe, Germany and purchased from Tianjin, China)
S4	Extract, extracted from tablets Ginaton® (made from EGb761 and manufactured by Dr. Willmar Schwabe GmbH &Co. KG, Karlsruhe, Germany and purchased from Tianjin, China)
S5	Extract of Ginkgo leaves collected from Shandong Province, China
S6	Extract of Ginkgo leaves collected from Hunan Province, China
S7	Extract, purchased from Ningbo Traditional Chinese Pharmaceutical Co., Ltd, Zhejiang Province, China
S8	Tanakan^®^ tablets made from EGb761 and manufactured by Beaufour Ipsen Industrie, Dreux, France and purchased from Watsons, HK
S9	Tanakan^®^ tablets made from EGb761 and manufactured by Beaufour Ipsen Industrie, Dreux, France and purchased from Tianjin, China
S10	Ginaton^®^ tablets made from EGb761 and manufactured by Dr. Willmar Schwabe GmbH &Co. KG, Karlsruhe, Germany and purchased from Tianjin, China
S11	Ginaton^®^ tablets made from EGb761 and manufactured by Dr. Willmar Schwabe GmbH &Co. KG, Karlsruhe, Germany and purchased from Tianjin, China

Samples S1–11 were subjected to UPLC-UV analysis to allow for the amounts of compounds **1**–**7** in these samples to be quantified. The results of this analysis (Table 4) revealed that similar levels of the seven FGs were present and very small batch-to-batch variation existed in the two commercial Ginkgo tablets Tanakan^®^ and Ginaton^®^, which reflected a high level of quality control during the manufacture of EGb761 and the tablets. In detail, the levels of the seven FGs in Tanakan^®^ were found to be a little higher than those in Ginaton^®^.

**Table 4:** FGs in the extracts and preparations of *Ginkgo biloba* (*n* = 2).

Compds.	Contents (µg/mg)								Contents (%) ^a^			
	S1	S2	S3	S4	S5	S6	S7		S8	S9	S10	S11
**1**	6.95	6.24	5.83	6.46	1.88	1.75	29.54		2.18	2.03	1.77	1.78
**2**	3.68	3.16	2.99	3.33	2.00	1.46	16.37		1.18	1.00	0.91	0.94
**3**	3.84	3.70	3.33	3.62	1.22	1.15	18.58		1.18	1.18	0.99	1.00
**4**	5.77	5.19	5.04	4.74	2.18	2.11	17.45		1.73	1.67	1.55	1.49
**5**	3.37	3.00	2.90	2.66	1.79	1.25	26.83		1.07	0.97	0.89	0.85
**6**	6.02	6.52	5.70	6.53	2.67	2.73	32.53		2.19	1.92	1.76	1.82
**7**	4.47	4.76	4.33	4.70	1.64	1.53	25.27		1.57	1.46	1.34	1.33
Sum	34.10	32.58	30.12	32.03	13.39	11.98	166.57		11.10	10.19	9.20	9.21

^a^: The amount of EGb761 per tablet was considered to be 40 mg based on the label for this preparation.

### 2.3. Determination of the Different TTLs using the HPLC-ELSD Method

#### 2.3.1. Calibration Curve

The linearity values of the TTLs **8**–**11** were determined by analyzing the curves of the four TTL standards at five different concentrations. The results were expressed as correlation coefficients (*R^2^*) and are summarized in Table 5.

**Table 5:** Linearity of the TTLs.

Compds.	Linearity (µg/mL)	Regression Equation	*R^2^*
**8**	36.725–734.50	logA = 1.58 logC + 1.44	0.9993
**9**	28.225–1129.0	logA = 1.51 logC + 1.54	0.9991
**10**	39.625–792.50	logA = 1.57logC + 1.38	0.9994
**11**	30.275–605.50	logA = 1.55 logC + 1.46	0.9996

#### 2.3.2. Quantitative Analysis of the Extracts and Preparations of *Ginkgo biloba*

The results of our quantitative analysis using USP protocol [9] indicated that there were specific differences between the two commercial Ginkgo tablets, Tanakan^®^ and Ginaton^®^, in terms of the amounts of the TTLs that they contained. For example, there were more of the four TTLs in Tanakan^®^ than there were in Ginaton^®^, although both products showed good batch-to-batch repeatability in their TTL contents (Table 6).

**Table 6:** TTLs in the extracts and preparations of *Ginkgo biloba* (*n* = 2).

Compds.	Contents (µg/mg)								Contents (%) ^a^			
	S1	S2	S3	S4	S5	S6	S7		S8	S9	S10	S11
**8**	5.99	6.23	4.94	5.52	5.02	3.51	42.04		2.17	2.11	1.78	2.04
**9**	14.57	14.78	11.73	11.84	5.59	4.59	53.23		1.00	1.09	0.82	1.00
**10**	6.00	6.21	6.09	6.08	6.14	4.10	13.57		2.15	2.14	1.63	1.70
**11**	14.73	13.3	10.94	10.88	5.37	4.18	32.98		3.23	3.22	2.63	2.72
Sum	41.28	40.52	33.69	34.31	22.11	16.36	141.82		8.55	8.55	6.86	7.46

^a^: The amount of EGb761 per tablet was considered to be 40 mg based on the label for this preparation.

### 2.4. Th-T Assay of the Main FGs and TTLs

The inhibitory activities of compounds **1**–**11** were evaluated using a Th-T assay. Myricetin, a flavonol which has been demonstrated to possess potent anti-amyloidogenic effects [10], was used as the positive control for the assay. Among the main FGs, three compounds (compounds **1**, **2**, and **6**) showed moderate and dose-dependent inhibitory activities towards Aβ42 fibril formation, with IC_50_ values in the range of 33 to 67 μM (Table 7). The other four FGs (compounds **3**, **4**, **5**, and **7**), however, showed weak effect (Table 8). Furthermore, even at very high concentrations (*i.e.*, 100 μM), the four main TTLs (compounds **8**–**11**) exhibited very little inhibitory activity towards Aβ42 fibril formation (Table 8).

**Table 7:** IC_50_ values of FGs **1**, **2** and **6** towards Aβ42 fibril formation.

Compds.	IC_50_ (μM)
**1**	33.02 ± 4.84
**2**	67.12 ± 11.66
**6**	32.56 ± 4.83
Myricetin	1.95 ± 0.32

**Table 8:** Inhibition rates (IRs) of compounds **3**–**5** and **7**–**11** towards Aβ42 fibril formation.

Compds. *	IR (%)
**3**	30.89 ± 3.90
**4**	23.62 ± 1.39
**5**	30.49 ± 1.36
**7**	34.98 ± 1.38
**8**	21.10 ± 2.96
**9**	13.56 ± 1.31
**10**	13.92 ± 2.35
**11**	14.84 ± 2.36

* All of the compounds were tested at a concentration of 100 μM.

Although our results for ginkgolides A, B, and C (**8**–**10**) were consistent with those reported in previous studies[11,12], our result for bilobalide (**11**) contrasted significantly with that reported by Luo *et al.* [12], who found that bilobalide exhibited significant *in vitro* activity towards the inhibition of Aβ40 fibril formation. The significant difference probably attributed to the different sequence between Aβ40 and Aβ42.

## 3. Experimental

### 3.1. Reagents and Materials

Ginkgolides A, B, and C, and bilobalide **8**–**11** (purity > 98%) were purchased from the National Institute for the Control of Pharmaceutical and Biological Products (Beijing, China). Catechin hydrate (purity ≥ 99%) and gallocatechin (purity ≥ 98%) were purchased from Nagara Science Co., Ltd. (Gifu, Japan). Compounds **1**–**7** were isolated in our laboratory and characterized by MS and NMR spectroscopy (purity > 95%). The Aβ42 peptide was produced by Bachem AG (Bubendorf, Switzerland) with purity greater than 95%, as determined by HPLC.

Thioflavin T (ThT), 1,1,1,3,3,3-hexafluoro-2-propanol (HFIP) and dimethyl sulfoxide (DMSO) were obtained from Sigma-Aldrich (St. Louis, MO, USA). Trifluoroacetic acid (TFA) was purchased from International Laboratory (South San Francisco, CA, USA). Tablets made from the extract of *Ginkgo* *biloba* leaves for isolation (distributed by General Nutrition Corporation, Pittsburgh, PA, USA) were purchased from the Manning pharmacy in Hong Kong.

Purifications by column chromatography were performed over Diaion HP-20 adsorbent resin (Mitsubishi Chemical Co., Tokyo, Japan), ODS (Davisil, 35–60 μm, Grace, Columbia, MD, USA), Sephadex LH-20 (Aldrich Chemical Co., Inc., Milwaukee, WI, USA) or Toyopearl TSK HW-40 gel (Tosoh Corporation, Tokyo, Japan). Silica gel 60 F_254_ HPTLC aluminium sheets (Merck, Darmstadt, Germany) were used for analysis by TLC.

### 3.2. Isolation, Preparation and Structural Elucidation of Acylated and Non-Acylated FGs from EGb761

The EGb761 tablets were ground into a powder, which was extracted with methanol for 1 h in an ultrasonic water bath, and this process was repeated three times. The combined extracts were filtered and concentrated under reduced pressure to give a residue, which was subjected to a Diaion HP-20 adsorbent resin column chromatography eluting with a gradient of methanol in water to afford 12 fractions.

Fraction 3 (1.2 g), which was eluted in 30% methanol in water (v/v), was stored in a refrigerator at 4 °C and gave a yellow precipitate. The precipitate was collected by filtration and re-crystallized from methanol to give compound **1** (10 mg) as an amorphous yellow powder.

Fraction 7 (2.3 g), which was eluted with 70% methanol in water (v/v), was purified by column chromatography over an ODS column eluting with a gradient of methanol in water to afford 12 sub-fractions. Sub-fractions 11 (0.1 g) and 13 (0.26 g) were chromatographed over TSK Toyopearl HW-40 gel eluting with a gradient of methanol in water to yield compounds **6** (22 mg) and **7** (52 mg), respectively. Sub-fraction 10 (0.49 g) was subjected to the same procedure to yield compounds **3** (25 mg), **4** (43 mg) and **5** (36 mg).

Compound **2** was prepared by the alkaline hydrolysis of compound **6** according to the procedure described below [13]. Compound **6** (15 mg) was dissolved in 0.4 mM solution of NaOMe in MeOH (0.5 mL), and the resulting solution was stirred in an ice bath for 3 h. Subsequent acidification with 0.3 N HCl, followed by concentration of the solution gave a residue, which was extracted with EtOAc. The EtOAc extract was then purified by column chromatography over ODS eluting with a gradient of methanol in water to give compound **2** (5 mg) as a yellow powder.

High resolution ESI-MS analysis was conducted on an Agilent 6230 time-of-flight mass spectrometer (Agilent, Santa Clara, CA, USA) in the positive ion mode. ^1^H- and ^13^C-NMR spectra were recorded at 600 and 150 MHz, respectively, on a Bruker CyroFIT NMR Spectrometer (Bruker Daltonics, Billerica, MA, USA).

### 3.3. Preparation of Extracts from Ginkgo Leaves and Products

Air-dried leaves of *Ginkgo biloba* was pulverized (over 80 mesh sieve) and the powder (5 g) was extracted twice with 60% methanol by sonication of 30 min at room temperature. After removal of the solvent by evaporation *in vacuo*, the extract was suspended in water and extracted twice with diethyl ether. The water layer was concentrated on a rotary concentrator and dried on a freeze drying system (Labconco, Kansas City, MO, USA) to obtain the extract of *Ginkgo* leaves.

Two commercial *Ginkgo* products made from EGb761, *i.e.*, Tanakan^®^ and Ginaton^®^ tablets, were removed of the coated film and grounded into powder. The powder was extracted twice with 60% methanol by sonication of 30 min at room temperature. The resulting extract was then concentrated and lyophilized on the freeze drying system to yield the extract of *Ginkgo* products.

### 3.4. Determination of FGs by UPLC-UV

Samples of *Ginkgo* extract (5 mg) were accurately weighed and transferred into a 2 mL tube followed by a solution of 60% methanol in water (v/v), and the resulting mixture was briefly shaken for mixing. The mixture was then sonicated for 10 min before being filtered through a 0.22 µm PTFE filter for analysis.

Samples of *Ginkgo* products, Tanakan^®^ and Ginaton^®^ tablets, were removed of the coated film and grounded into powder. The powder (equivalent to 40mg EGb761) was extracted twice with 60% methanol by sonication of 30 min at room temperature. The supernatant was transferred into a 25 mL volumetric flask and made up to its volume, and then filtered through a 0.22 µm PTFE filter for analysis.

The reference compounds were accurately weighed and dissolved in a mixture of 60% methanol in water (v/v) to prepare a standard mixed stock solution containing compounds **1** (300.0 µg/mL), **2** (144.0 µg/mL), **3** (150.0 µg/mL), **4** (150.0 µg/mL), **5** (150.0 µg/mL), **6** (150.0 µg/mL) and **7** (150.0 µg/mL). Working standard solutions for the calibration curves were prepared by diluting the mixed standard solutions with a mixture of 60% methanol in water (v/v) to the appropriate concentrations. The standard solutions were then stored at 4 °C prior to being analyzed.

UPLC was performed on an Acquity UPLC system (Waters Corp, Milford, MA, USA) equipped with a binary solvent delivery system, auto-sampler unit, and diode array detector (DAD). All the operations, including the acquisition and analysis of data were controlled using Hystar software (Bruker). The chromatography was performed on an Acquity BEH C18 column (2.1 × 100 mm, 1.7 μm). The mobile phases consisted of 0.1% formic acid in water (A) and 0.1% formic acid in acetonitrile (B).

The following gradient elution procedure was used for the analysis of the sample: 0–2 min, 5%–15% B; 2.1–20 min, 15%–22% B; 20.1–23 min, 100% B; 23.1–25 min, 5% B. For quantitative analysis, the flow rate was set at 0.35 mL/min, the column temperature was kept at 40 °C, the detector signal was set at 260 nm, and the injection volume was set at 2 µL.

A calibration curve was established for the quantitative analysis of the flavonoids by plotting the peak areas of analytes against the concentrations of the standard solutions, and then the amounts of the different FGs in the samples were then calculated using the calibration curve.

### 3.5. Determination of TTLs using a HPLC-ELSD Method

Standard and test solutions were prepared and analyzed according to the procedure described for USP28-NF23 [9]. The standard solutions were prepared as follows: accurately weighed quantities of ginkgolides A, B and C and bilobalide were dissolved in methanol, and the resulting solutions were sonicating for 5 min before being diluted with methanol to obtain solutions of different concentrations, including 0.1, 0.5, 1.0, 2.0, 4.0, and 8.0 mg/mL. The solutions were then passed through a 0.45 µm filter.

The test solutions were prepared as follows: An accurately weighed portion of the *Ginkgo* sample was placed in a 30 mL glass tube with screw cap and PTFE gasket followed by 10.0 mL of a 90% methanol solution. The tube was then sealed, and the mixture heated with stirring at 90 °C for 30 min. The hot suspension was then mixed on a vortex mixer for 5 min before being heated at 90 °C for 30 min. The mixture was then cooled to room temperature, and passed through a 0.45 µm filter.

The mixtures were analyzed by HPLC using a Waters 2695 HPLC system equipped with a Waters 2420 evaporative light scattering detector (ELSD) (Waters Corp). A Phenomenex C18 column (250 × 4.60 mm, 5 µm) was used for the chromatography eluting with mobile phases consisting of water (A) and MeOH (B).

The following gradient elution procedure was used for the analysis: 0–23 min, 25%–48% B; 23–25 min, 48%–75% B; 25–30 min, 75% B; 30–35 min, 75%–90% B; 35–40 min, 90%–25% B; 40–50 min, 25% B.

The HPLC system was operated with a flow rate of 1.0mL/min with column and drift tube temperatures of 30 and 55 °C, respectively. The nebulizer temperature was set at 36 °C and the gas pressure was 25 psi. The injection volume for the quantitative analysis was set to 10 µL.

### 3.6. Preparation of Aβ Stock Solutions

The Aβ stock solutions were prepared according to a simple TFA pre-treatment protocol [14]. The Aβ42 peptide was suspended in TFA at a concentration of 2 mg/mL, and the resulting mixture was sonicated for 20 min at room temperature to allow for facilitate complete dissolution. A gentle stream of nitrogen gas was passed over the mixture to remove the TFA, and the resulting residue was treated with HFIP to give a suspension, which was cooled on ice without agitation until the peptide dissolved completely. The HFIP was then removed by evaporation with a gentle stream of nitrogen gas. The resulting peptide was lyophilized overnight on a freeze drying system (Labconco) to remove any trace amounts of TFA and HFIP, and then dissolved in cold 0.02% ammonia solution to a final concentration of 250 μM and stored at −80 °C before assay.

### 3.7. Polymerization Assay

A polymerization assay was performed in the current study using a previously described procedure [15,16]. A reaction mixture containing 25 μM Aβ42 was mixed with different amounts (*i.e.*, 0.3, 1.0, 3.0, 10, 30, 50 and 100 μM) of compounds **1**, **2** and **6**, as well as 1% DMSO, 50 mM phosphate buffer (pH 7.5) and 100 mM NaCl. All of compounds for testing were initially dissolved in DMSO at concentrations of 30, 100, and 300 μM, and 1, 3, 5 and 10 mM, and then added to the reaction mixture to final concentrations of 0.3, 1.0, 3.0, 10, 30 and 50 μM, respectively. Compounds **3**, **4**, **5**, **7**, **8**, **9**, **10** and **11** showed very low levels of activity in our preliminary activity test and were subsequently tested at a final concentration of 100 μM in the full assay.

Twenty microliter aliquots of the mixtures were injected into PCR tubes, which were then placed into the PCR system (Gene Amp PCR System 9700, Applied Biosystems, Foster City, CA, USA) and incubated of 24 h without agitation. The reactions were stopped by adding a ThT solution. Fluorescence measurements were conducted in triplicate from every tube with the mean values being reported. The concentrations of test compounds in the testing solutions were diluted to 1/150 of those in the reaction solution to minimize any quenching effects.

### 3.8. Fluorescence Spectroscopy

Fluorescence spectroscopy was conducted on an Envision 2104 Multilabel Reader (Perkin Elmer, Waltham, MA, USA), with optimized excitation and emission wavelengths of 430 (BW 24 nm) and 470 nm (BW 24 nm), respectively. The reaction mixtures contained 5 μM ThT and 50 mM of glycine-NaOH buffer (pH 8.5). IC_50_ values were calculated using the GraphPad Prism 5 software.

## 4. Conclusions

Seven major FGs, including two characteristic components **6**, **7** were isolated from the extract of *Ginkgo* *biloba* leaves and their structures were identified on the basis of MS and NMR spectral evidences. A UPLC-UV method was developed for the simultaneous determination of these flavonoids in Ginkgo extract and products. The linearity, LOD, LOQ, precision, repeatability and recovery characteristics of this method revealed that it was suitable for the quantitative analysis of the FGs in EGb761. Regarding the inhibitory activity of the pure compounds towards Aβ fibril formation, significant differences were observed between these seven compounds. For example, compounds **1**, **2** and **6** exhibited moderate inhibitory activities towards Aβ42 fibril formation, whereas compounds **3**, **4**, **5** and **7** showed barely any effect. We also confirmed that four TTLs exhibited no inhibitory activity towards Aβ42 fibril formation. This study therefore represents the first reported example of the isolation of acylated flavonoid glycosides **6**, **7** from EGb761 with inhibitory activity against Aβ fibril formation. Given that FGs are one of the major components of EGb761, it is reasonable to indicate that these constituents have contributed in part to the neuroprotective effect of EGb761. Furthermore, the results of the current study have provided some structure-activity relationships for the FGs in terms of inhibitory activity towards Aβ42 fibril formation. Of the seven FGs evaluated in the current study, the three FGs **1**, **2**, **6** with the aglycone of quercetin showed much stronger activity than those with the aglycone of kaempferol or isorhamnetin, indicating that the observed activity was closely associated with the aglycone moiety, and that the number and position of the hydroxyl groups could have a significant impact on the activity. It is reasonable to indicate that the moiety of two adjacent hydroxide groups in B ring is necessary for these glycosides to exert significant inhibitory effect on Aβ42 fibril formation.

The difference observed between the activities of compounds **1** and **2** was attributed to the linkage of the sugar moiety. It is noteworthy that acylated compound **6** showed much stronger inhibitory activity than **2**. This difference in activity was attributed to the three-dimensional conformation formed as a consequence of the coumaroyl group in **6**. This conformation is essential for the compound to form a non-covalent interaction with the β-sheet structure of the amyloid peptide [8,17].

In conclusion, we have shown that seven FGs from the extract of the leaves of *Ginkgo* *biloba* showed moderate to weak inhibitory activity towards Aβ42 fibril formation, whereas four TTLs had little effect. However, it is important to mention that these effects were much less pronounced than that of EGb761 in an equivalent dose, suggesting that other additional unknown compounds present in EGb761 must be responsible for its inhibitory activity on Aβ42 fibril formation. At this point, polyphenols existed in EGb761 might be another category of constituents contributing to the neuroprotective effect of EGb761 as potential activities of EGCG have been suggested [18,19].

## References

[1] Cavallucci V., D’Amelio M., Cecconi F. (2012). Aβ Toxicity in Alzheimer’s Disease. Mol. Neurobiol..

[2] Savonenko A.V., Melnikova T., Hiatt A., Li T., Worley P., Troncoso J.C., Wong P.C., Price D.L. (2012). Alzheimer’s Therapeutics: Translation of Preclinical Science to Clinical Drug Development. Neuropsychopharmacogy.

[3] Cott J.M., Raman A., Herabalist R.U., Graff A. (2003). Clinical Efficacy and Pharmacodynamics. American herbal Pharmacopoeia and Therapeutic Compendium: Ginkgo Leaf, Ginkgo Leaf Dry Extract, Ginkgo biloba L..

[4] Levine H. (1993). Thioflavine T interaction with synthetic Alzheimer’s disease β-amyloid pepetides: Detection of amyloid aggregation in solution. Prot. Sci..

[5] Nasr C., Lobstein-Guth A., Haag-Berrurier R.A.A. (1987). Quercetin coumaroyl glucorhamnoside from Ginkgo biloba. Phytochemistry.

[6] Nasr C., Haag-Berrurier R.A., Lobstein-Guth A., Anton R. (1986). Kaempferol coumaroyl glucorhamnoside from *Ginkgo biloba*. Phytochemistry.

[7] Tang Y.P., Wang Y., Lou F.C., Li Y.F., Wang J.H. (2000). Flavonol glycosides from the leaves of Ginkgo biloba. Acta Pharm. Sin..

[8] Gao J.H., Shi F.G., Song G.Q. (1996). Further NMR investigation and conformational analysis of an acylated flavonol glucorhamanoside. Magn. Reson. Chem..

[9] The United States Pharmacopeial Convention (2003). The United States Pharmacopeia, 28th Revision/National Formulary.

[10] Ono K., Yoshiike Y., Takashima A., Hasegawa K., Naiki H., Yamada M. (2003). Potent anti-amyloidogenic and fibril-destabilizing effects of polyphenols in vitro: implications for the prevention and therapeutics of Alzheimer’s disease. J. Neurochem..

[11] Ramassamy C., Christen Y., Poirier J., Christen Y. (2001). *Ginkgo biloba* extract (EGb761), β-amyloid peptide and apolipoprotein E in Alzheimer’s disease. Advances in Ginkgo biloba Extract (EGb761) as a Neuroprotective Agent: From Basic Studies to Clinical Trial.

[12] Luo Y., Smith J.V., Paramasivam V., Burdick A., Curry K.J., Buford J.P., Khan I., Netzer W.J., Xu H.X., Butko P. (2002). Inhibition of amyloid-β aggregation and caspase-3 activation by the *Ginkgo biloba* extract EGb761. Natl. Acad. Sci. USA.

[13] Jiang Z.-H., Tanaka T., Kouno I. (1996). Chilianthins A-F, six triterpene esters having dimeric structures from Rhoitelea chiliantha DIELS et HAND.-MAZZ. Chem. Pharm. Bull..

[14] Jao S.C., Ma K., Talafous J., Orlando R., Zagorski M.G. (1997). Trifluoroacetic acid pretreatment reproducibly disaggregates the amyloid β-peptide. Amyloid Int. J. Exp. Clin. Invest..

[15] Naiki H., Hasegawa K., Yamaguchi I., Nakamura H., Gejyo F., Nakakuki K. (1998). Apolipoprotein E and antioxidants have different mechanisms of inhibiting Alzheimer’s beta-amyloid fibril *in vitro*. Biochemistry.

[16] Ono K., Hasegawa K., Yamada K., Naiki H. (2002). Nicotine breaks down performed Alzheimer’s beta-amyloid fibril *in vitro*. Biol. Psychiatry..

[17] Porat Y., Abramowitz A., Gazit E. (2006). Inhibition of amyloid fibril formation by polyphenols: Structural similarity and aromatic interactions as a common inhibition mechanism. Chem. Biol. Drug. Des..

[18] Zhang H.Y., Wang J.R., Yau L.F., Ho H.M., Chan C.L., Hu P., Liu L., Jiang Z.H. (2012). A cellular lipidomic study on the Aβ-induced neurotoxicity and neuroprotective effects of EGCG by using UPLC/MS-based glycerolipids profiling and multivariate analysis. Mol. Biosyst..

[19] Bai L.P., Ho H.M., Ma D.L., Yang H., Fu W.C., Jiang Z.H. (2013). Aminoglycosylation can enhance the G-quadruplex binding activity of epigallocatechin. PLoS One.

